# Mushrooms as Rainmakers: How Spores Act as Nuclei for Raindrops

**DOI:** 10.1371/journal.pone.0140407

**Published:** 2015-10-28

**Authors:** Maribeth O. Hassett, Mark W. F. Fischer, Nicholas P. Money

**Affiliations:** 1 Department of Biology, Miami University, Oxford, Ohio 45056, United States of America; 2 Department of Chemistry and Physical Science, Mount St. Joseph University, Cincinnati, Ohio 45233, United States of America; 3 Western Program, Miami University, Oxford, Ohio 45056, United States of America; Dartmouth College, UNITED STATES

## Abstract

Millions of tons of fungal spores are dispersed in the atmosphere every year. These living cells, along with plant spores and pollen grains, may act as nuclei for condensation of water in clouds. Basidiospores released by mushrooms form a significant proportion of these aerosols, particularly above tropical forests. Mushroom spores are discharged from gills by the rapid displacement of a droplet of fluid on the cell surface. This droplet is formed by the condensation of water on the spore surface stimulated by the secretion of mannitol and other hygroscopic sugars. This fluid is carried with the spore during discharge, but evaporates once the spore is airborne. Using environmental electron microscopy, we have demonstrated that droplets reform on spores in humid air. The kinetics of this process suggest that basidiospores are especially effective as nuclei for the formation of large water drops in clouds. Through this mechanism, mushroom spores may promote rainfall in ecosystems that support large populations of ectomycorrhizal and saprotrophic basidiomycetes. Our research heightens interest in the global significance of the fungi and raises additional concerns about the sustainability of forests that depend on heavy precipitation.

## Introduction

**Fig 1:**
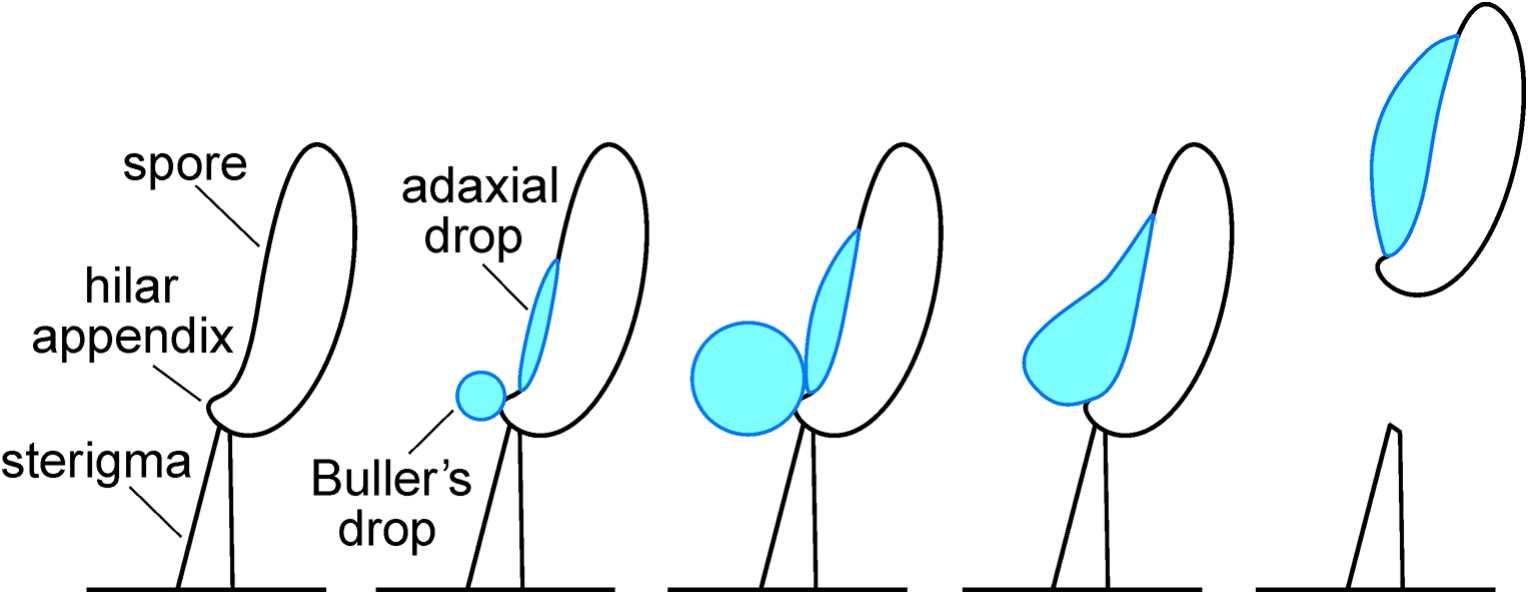
Schematic showing process of ballistospore discharge. Buller’s drop and the adaxial drop form via condensation of water on the spore surface and their coalescence causes a rapid shift in the center of mass of the spore that is responsible for the launch.

Million of tons of fungal spores are dispersed in the atmosphere every year [1]. An individual gilled mushroom can release 30,000 basidiospores every second, corresponding to a daily output of billions of microscopic particles [2]. Basidiospores are discharged from the gill surfaces by a catapult mechanism powered by the rapid movement of a drop of fluid over the spore surface (Fig 1). This fluid is called Buller’s drop in tribute to “the Einstein of Mycology,” A. H. R. Buller (1874–1944). Buller’s drop is formed by the condensation of water on the spore surface that is stimulated by the secretion of mannitol and other hygroscopic sugars [3, 4]. Water also condenses on a spot on the adjacent spore surface. The merger of Buller’s drop with this second volume of fluid (the adaxial drop) causes a rapid displacement of the center of mass of the spore. This fluid motion, driven by surface tension, imparts momentum to the spore and it is launched at an initial velocity of up to 1.8 m s^-1^ [5]. This mechanism was proposed in some detail in the 1980s [6], and verified later by high-speed video recordings [7].

Discharged spores fall in the narrow spaces between the gills and are dispersed in airflow around the mushroom cap. The same discharge mechanism operates in poroid mushrooms, with fertile tubes rather than gills, in mushrooms with spines, and from the great diversity of fruit bodies that form spores on exposed surfaces. Fluid is carried with basidiospores after discharge, but evaporates once the spore is airborne. Mushroom spores are a primary source of mannitol detected in air samples above tropical forests [1]. Using mannitol as a biotracer, Elbert et al. (2007) estimated that 50 million tonnes of spores are dispersed in the atmosphere every year. This biomass is carried by an Avogadroian number of spores, corresponding to an average of 1,000 spores for every square millimeter of Earth’s surface. Other biogenic aerosols include plant spores and pollen grains. These “primary biological aerosol particles” appear to be particularly important in the Amazon Basin, and other densely forested locations, where they may serve as nuclei for cloud formation and precipitation [8]. Details of the hygroscopic behavior of mushroom spores have not been studied previously.

Environmental scanning electron microscopy (ESEM) has been used in a number of experiments to study the condensation of water on pollen and other particles that become aerosolized [9–13]. This technique allows investigators to study the properties of untreated biological samples to preserve natural surface chemistry and to visualize the condensation of water in real time. In the present study, we report ESEM experiments showing that the extraordinary mechanism of spore discharge in mushrooms has a specific effect on water condensation after spores are dispersed in the atmosphere. This suggests that mushroom spores are particularly powerful catalysts for raindrop formation in clouds and contribute to rainfall. This may be an important phenomenon in ecosystems that support large populations of ectomycorrhizal and saprotrophic basidiomycetes. Our research heightens interest in the global significance of the fungi and raises additional concerns about the sustainability of forests that depend on heavy rainfall.

## Materials and Methods

### Fungal specimens

**Fig 2:**
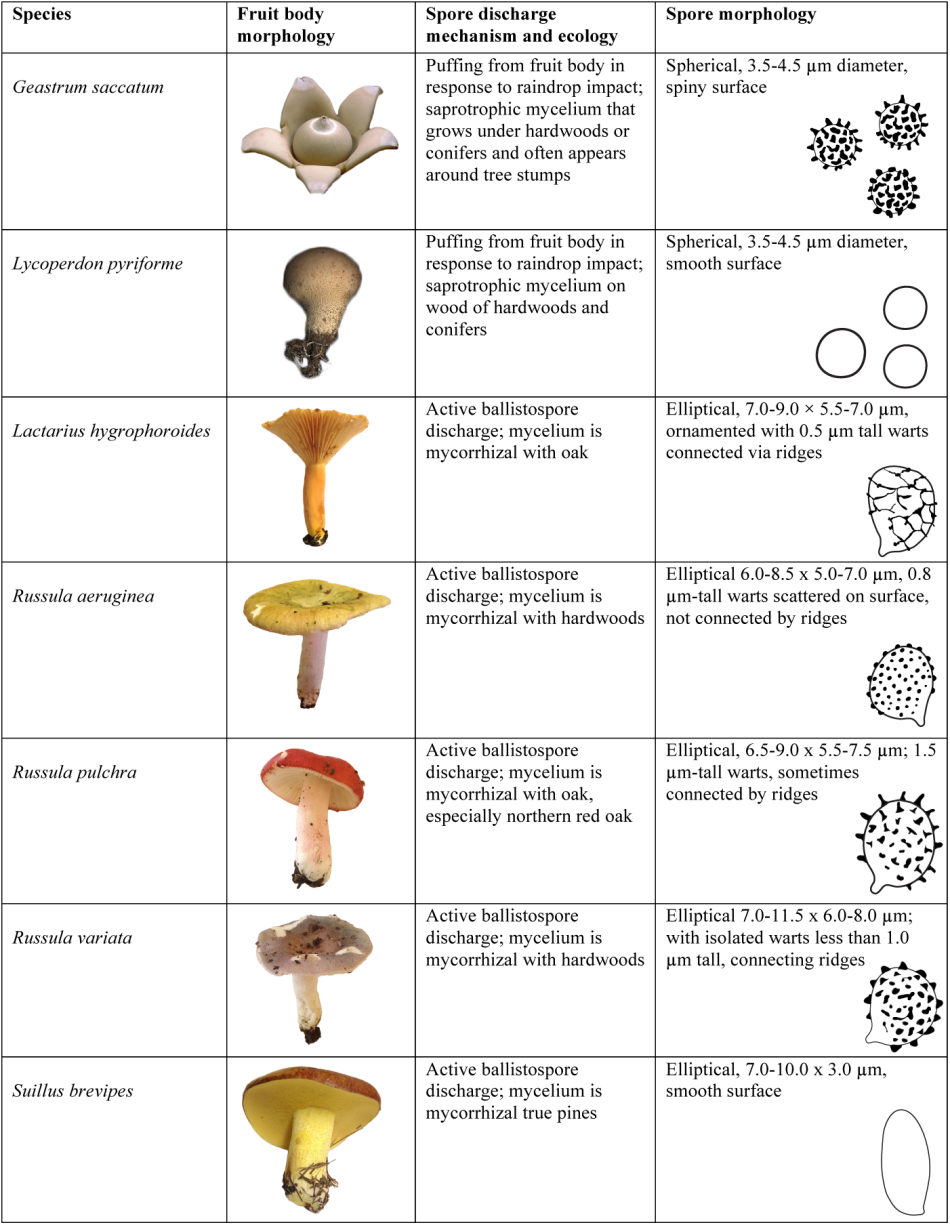
Species of basidiomycete fungi (Class Agaricomycetes) examined in this study.

Fresh specimens of basidiomycete fruit bodies were collected from the Miami University Natural Areas in Oxford, Ohio (Fig 2). Permits are not required for collecting specimens from these woodlands and none of the fungi are listed as protected or endangered species. These species included gilled and poroid mushrooms whose spores are discharged via the drop mechanism (species of *Lactarius*, *Russula*, and *Suillus*), and a puffball (*Lycoperdon pyriforme*) and earth-star (*Geastrum saccatum*) whose spores are expelled from the fruit body by the impact of raindrops. Gilled and poroid mushrooms were positioned above ESEM aluminum stubs to allow a fine deposit of spores to accumulate for 1–4 h. The puffball and earth-star spores were deposited on the ESEM stubs by gentle squeezing of the fruit bodies to cause puffing. The spores were not treated with any chemicals prior to imaging.

### Environmental Scanning Electron Microscopy (ESEM)

Samples were analyzed using an environmental scanning electron microscope (FEI Quanta 200 ESEM, Hillsboro, OR). Non-coated stubs carrying the spore deposits were mounted on a Peltier cooling stage inside the microscope and the temperature was stabilized at 3 C. The relative humidity inside the sample chamber was controlled by maintaining a constant temperature and altering the vapor pressure (Hiranuma et al. 2008). This provides conditions of supersaturation inside the specimen chamber according to the Clausius-Clapyron equation [14]:

$$RH=\frac{P}{P_{S}∙exp(\frac{L_{V}}{R_{V}}∙(\frac{1}{273.15}-\frac{1}{T_{C}+273.15}))}∙100\%$$

*RH* = relative humidity

*P* = pressure in torr

*P*_*S*_ = saturated vapor pressure at 273.15°C = 4.58 torr

*L*_*V*_ = latent heat of vaporization of water = 2.26 x 10^6^ J kg^-1^

*R*_*V*_ = Gas constant for water vapor = 461.5 J/(kg K)

*T*_*C*_ = temperature in °C

For the analysis of hygroscopic behavior, each spore deposit of each species was subjected to 2–4 repetitions of identical changes relative humidity.

## Results

### Dynamics of drop formation

**Fig 3:**
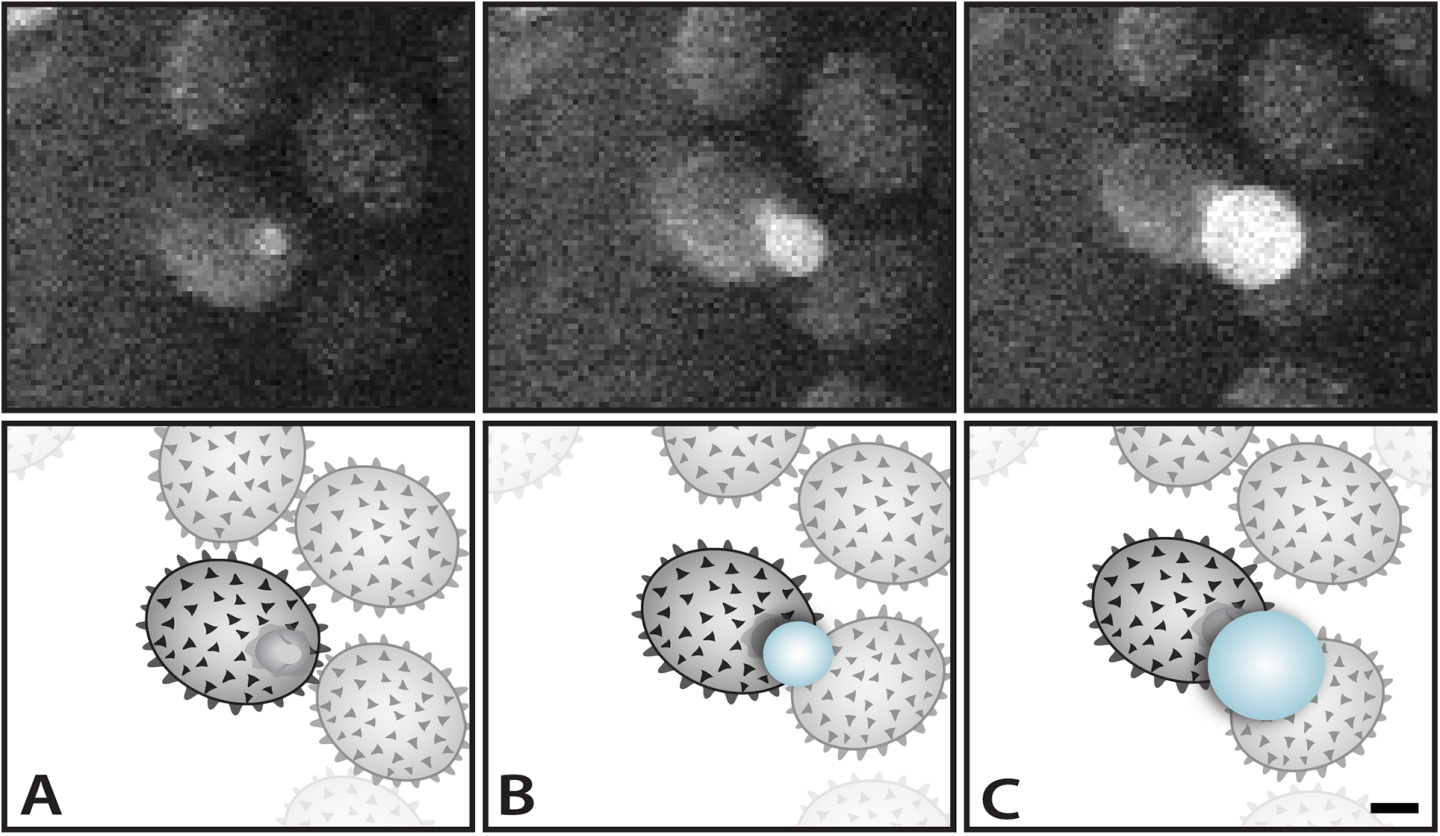
ESEM images (A-C) and accompanying diagrams (D-F) showing droplet formation at 102% RH on the hilar appendix of a basidiospore dispersed from *Russula aeruginea*. (A, D) Spore prior to droplet formation with hilar appendix appearing as rounded protruberance from the base of the spore. (B, E) Droplet beginning to form, and (C, F) continuing to expand. Scale = 2 μm.

**Fig 4:**
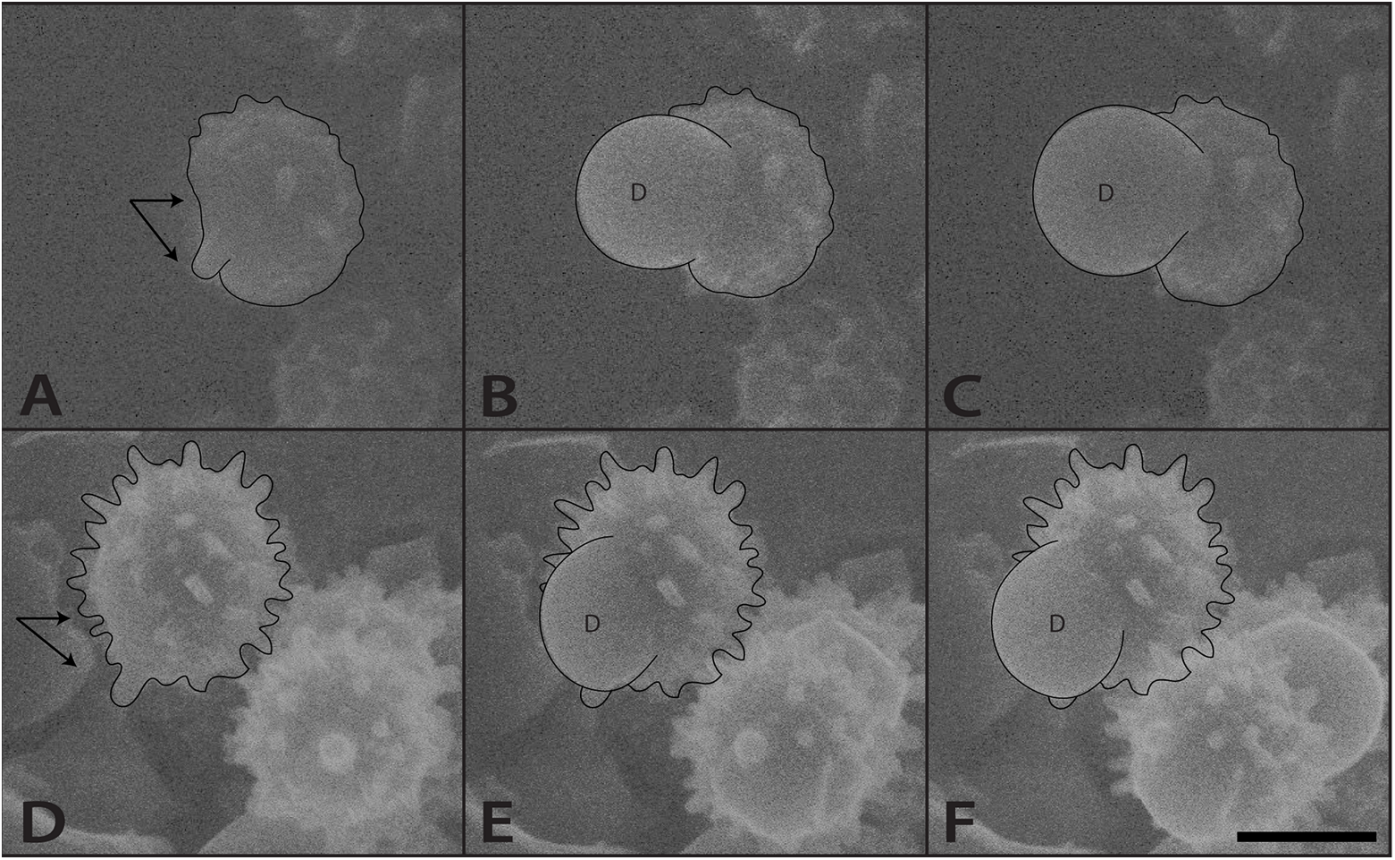
ESEM images of droplet (d) formation on the adaxial surface of basidiospores. (A-C) *Lactarius hygrophoroides*, (D-F) *Russula pulchra*. The spores in this figure are viewed in profile with the hilar appendix protruding from the base and the adaxial surface above (arrows). (A-C) 103% RH; (D) 98% RH; (E,F) 102% RH. Scale = 5 μm.

Spores with prominent hilar appendices from gilled and poroid mushrooms were studied under conditions of increasing relative humidity (RH) using the ESEM. Water droplets developed on the hilar appendix (Fig 3) and on the adaxial surface (Fig 4) of these spores between relative humidities of 101% and 102%. Image sequences were captured showing the spore surface prior to droplet initiation, formation of the initial droplets, and subsequent expansion of droplets (Figs 3 and 4, S1 Video and S2 Video). These experiments show that Buller’s drop and the adaxial drop can reform on the spore after discharge.

**Fig 5:**
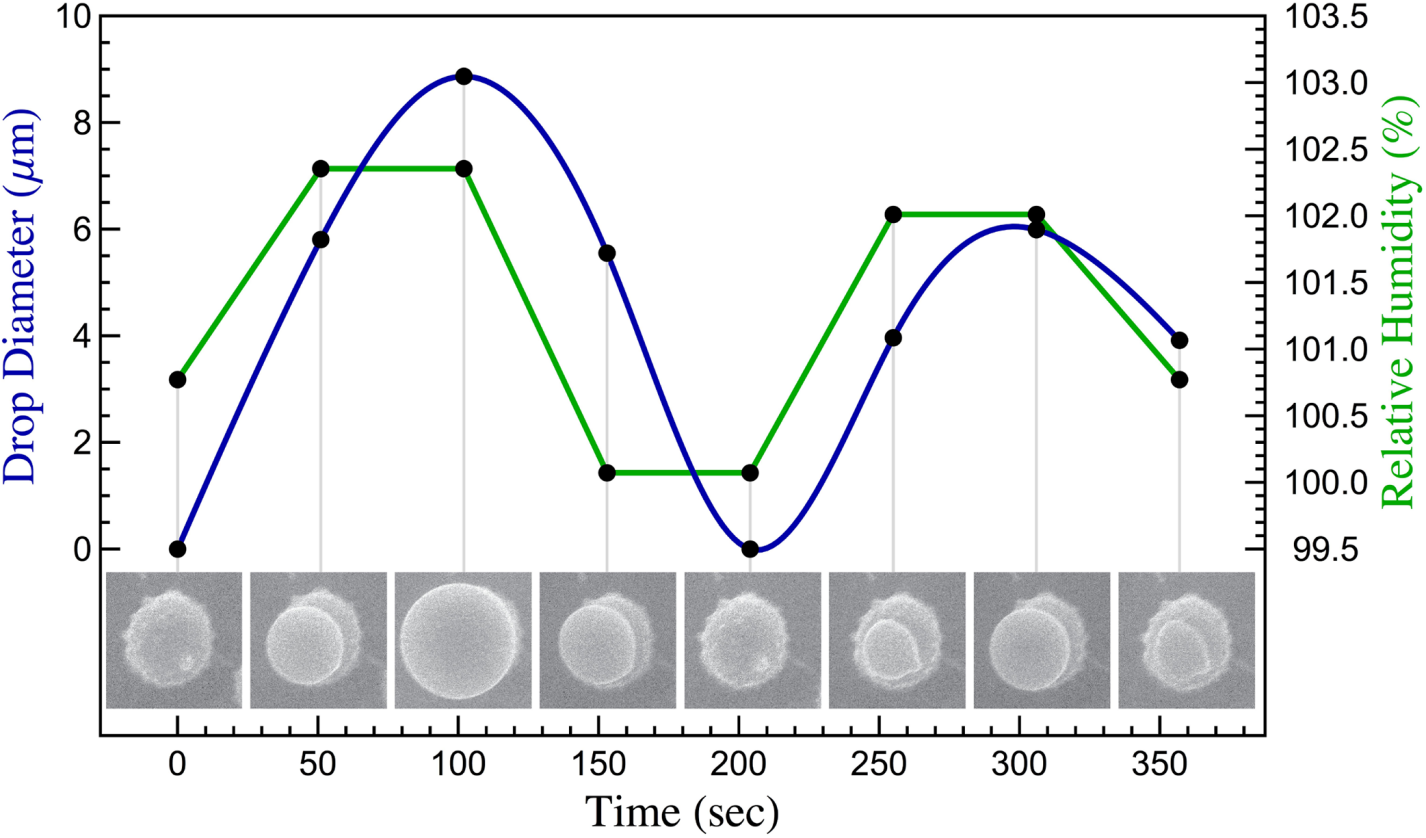
Dynamics of droplet growth, evaporation, and reformation on the surface of a basidiospore of *Russula variata* in response to changes in relative humidity controlled in the ESEM.

The sensitivity to drop expansion on the hilar appendix and adaxial surface of the spores was demonstrated by controlling the RH inside the sample chamber by increasing or decreasing the water vapor pressure within the sample chamber of the microscope (Fig 5). Water condensing on the spore forms large droplets when RH is increased to 102% RH, and shrinks when it is dropped below 100% RH, appearing as a thin film on the spore surface before complete evaporation. New drops were generated on the same spores after multiple rounds of dehydration and rehydration.

**Fig 6:**
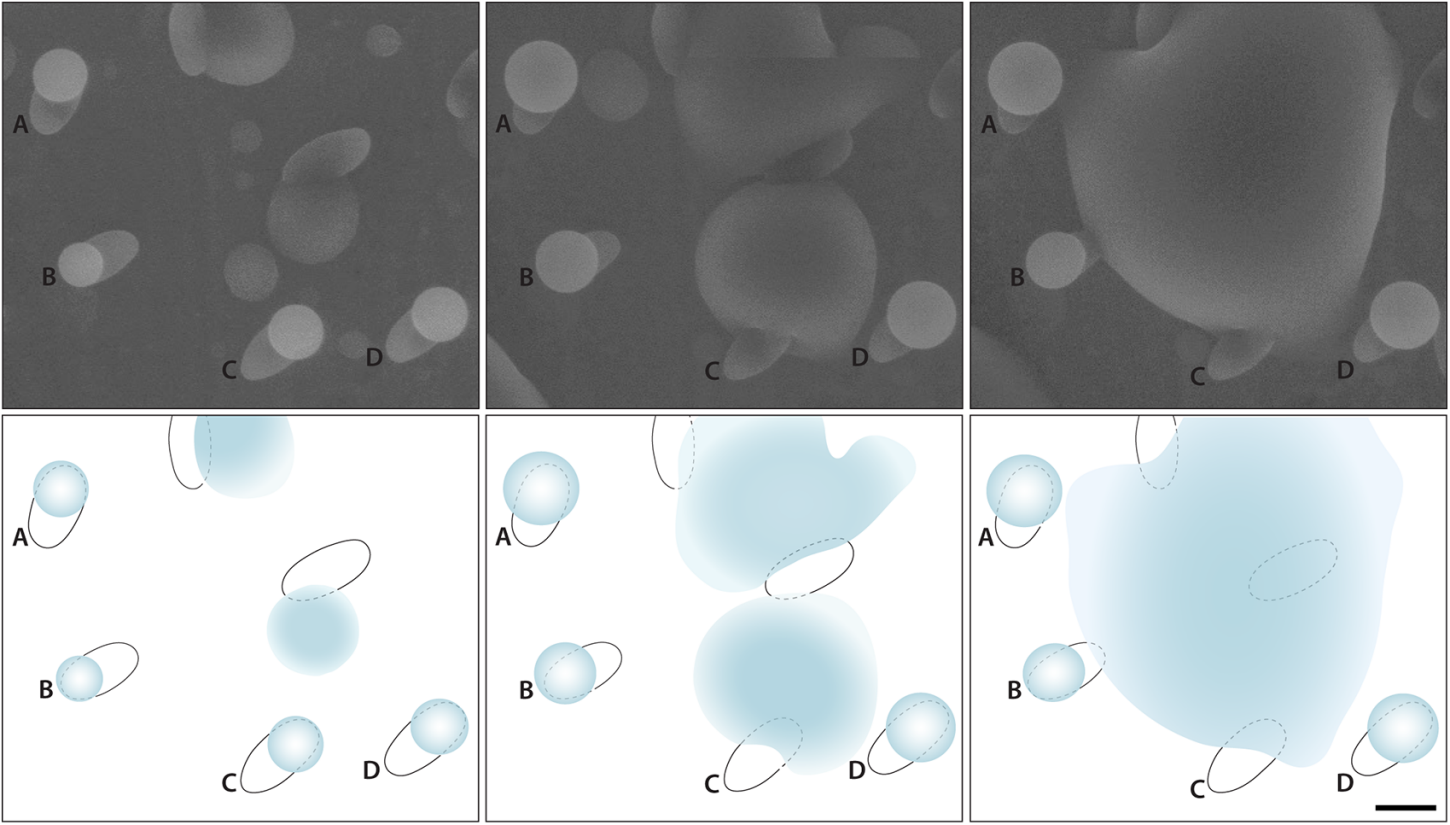
Droplet formation on basidiospores of *Suillus brevipes* in the ESEM at 101% RH. (A-D) Spores are orientated with adaxial surface facing away from surface of the specimen stub, allowing observation of droplets growing from this hygroscopic region of the spore. Note merger of droplets in second and third panels. Scale = 5 μm.

Several factors dictate the maximum size of Buller’s drop and the adaxial drop during spore discharge. Buller’s drop is constrained by spore size and geometry, hydrophobicity of spore surface, and the growth of the adaxial drop with which it fuses [5,15]. In the ESEM experiments, droplets were observed growing from the hilar appendix, and from the adaxial surface, but not from both parts of the same spore surface simultaneously. The maximum size of droplets growing from the hilar appendix of spores is comparable to those predicted from spore dimensions [5,15] and associated with normal spore discharge (Fig 3). Droplets growing from the adaxial surface of spores became much larger, with the diameter of droplets often exceeding the diameter of the spore (Fig 6). The largest drops associated with single spores expanded to a diameter of 13 μm and 27 μm drops surrounded clusters of three spores.

### Behavior of non-ballistosporic basidiospores

**Fig 7:**
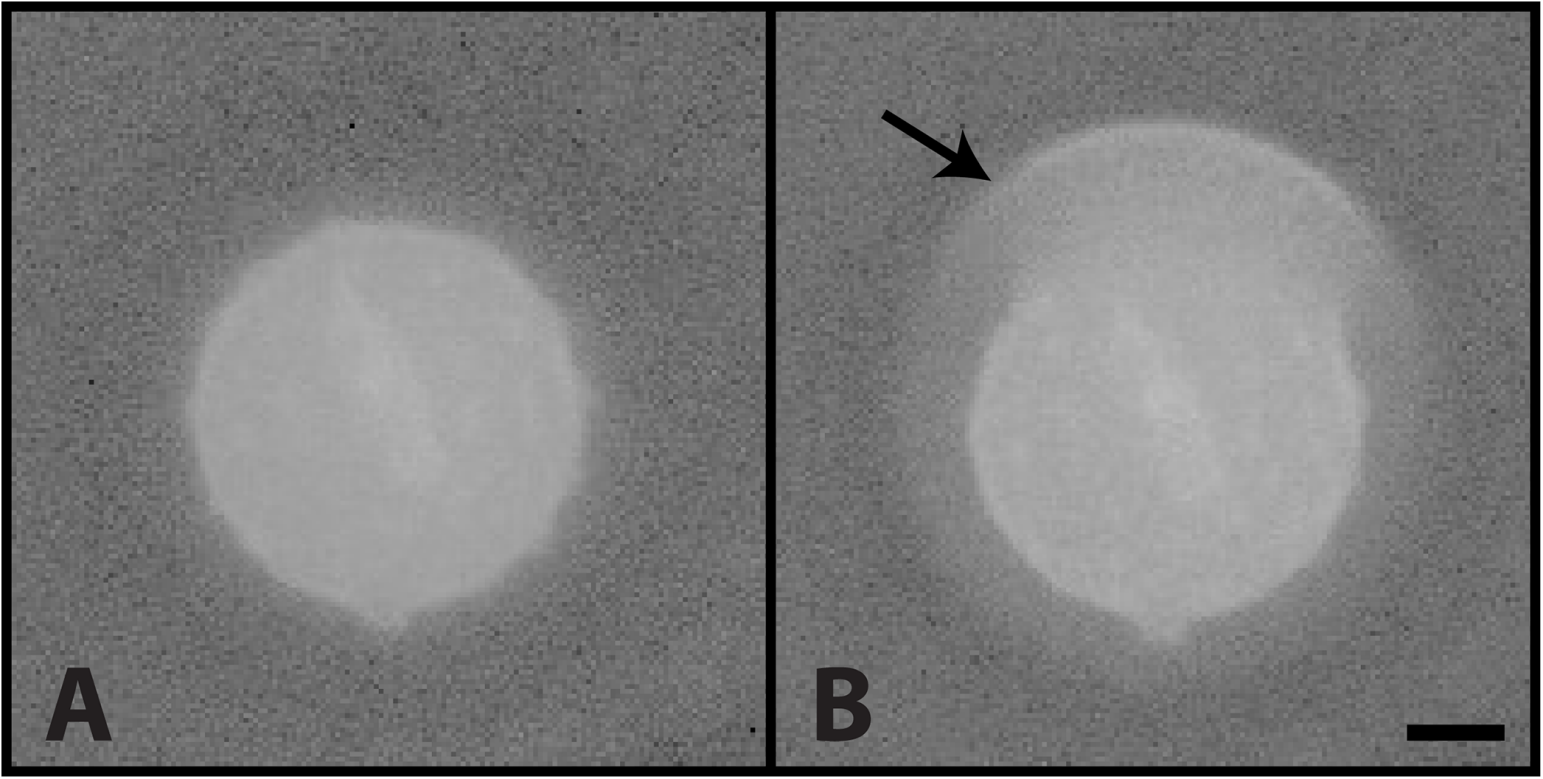
Condensation of water on non-ballistosporic basidiospore from the puffball, *Lycoperdon pyriforme*. (A) Spore prior to condensation of water at 100% RH and (B) with halo of water surrounding the spore at 101% RH. Scale = 2 μm.

There were significant differences in the pattern of water condensation on the surface of non-ballistosporic basidiospores of *Lycoperdon pyriforme* and *Geastrum saccatum*. As the vapor pressure in the ESEM specimen chamber was increased to boost the RH from 100% to 102%, liquid water began to accumulate on the spores, but formed a thin shell around the entire spore rather than expanding as discrete droplets. This accumulation of water was visible as a thin halo around each spore (Fig 7). This behavior contrasts with the expansion of droplets on the surface of ballistospores that often exceeded the size of the spores.

## Discussion

### Drop growth

Drop expansion on the hilar appendix of spores in the ESEM resembles the process that creates the Buller’s drop in the seconds before spore discharge from mushroom gills (Fig 3). The regrowth of this discrete drop is only visible in spores that are oriented with the hilar appendix pointing away from the surface of the stub on which the spores are deposited. Drop formation in the ESEM on the adjacent spore surface is more common (Fig 4). This may be explained by the coalescence of the two droplets on the spore at the moment of spore discharge (Fig 1). When this occurs, mannitol secreted on the hilar appendix and adjacent surface is mixed in the combined volume of water (Fig 1). When water evaporates from the spore surface, the mannitol is spread over a larger portion of the spore surface. This would explain why the water that condenses on the spores grows as a single drop rather than two separate drops. Because the spores in the ESEM do not move, water vapor continues to condense on their surfaces creating drops that are significantly larger than those that form during the discharge process.

### Significance in clouds

The dispersal of vast numbers of hygroscopic basidiospores into the atmosphere above forests may have a significant role in the condensation of water in clouds and the formation of raindrops. Previous studies have suggested that fungal spores may operate as ice-nucleating agents at low temperatures [16]. We propose a different role for basidiospores in the dynamics of cloud formation and rainfall in the present study. Supersaturation is a transient and dynamic state in clouds in which the moisture levels exceed a RH of 100%. Typical levels of supersaturation in clouds boost RH values to 102% [17] and rarely exceeds 110% RH [18]. Droplet formation on spores in the ESEM observed at RH values ranging from 100% to 102% RH match the environmental conditions found in clouds.

**Fig 8:**
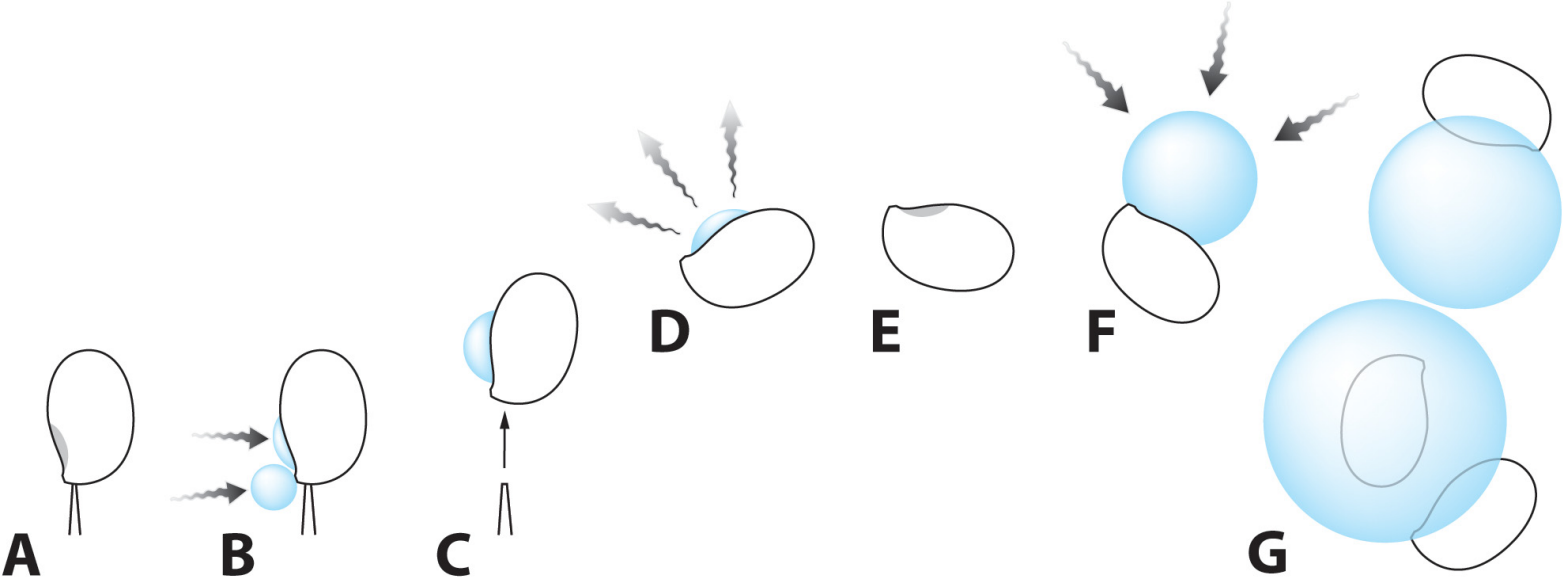
Overview of the mechanism of droplet formation on the surface of basidiospores. (A-C) Condensation of water on the spore surface associated with spore discharge. Process of drop formation is driven by the presence of hygroscopic sugars on the spore surface in the two positions shown in gray in A. (D, E) Water evaporates from the surface of the airborne spore. (F) Condensation of water on the spore surface resumes under conditions of high atmospheric relative humidity. (G) Larger droplets of water formed by merger of spores carrying smaller droplets.

Coarse aerosolized particles with a diameter exceeding 2 μm play an important role within clouds as seeds for the formation of water droplets. When water condenses on the surface of these particles, they operate as giant cloud condensation nuclei (GCCN) that form larger droplets by coalescing with smaller droplets and merging with one another [19]. Raindrop formation occurs via coalescence of water droplets with GCCN rather than sustained condensation of water vapor [20]. Hygroscopic basidiospores, which are classed as large aerosol particles, may be particularly effective as GCCN. The size of the droplets observed in the ESEM suggest that mushroom spores act as giant cloud condensation nuclei, aiding the coalescence of smaller droplets to form precipitation-sized drops (Fig 8). Non-ballistosporic basidiospores may act also as nuclei for water condensation in clouds, but they operate in a similar fashion to particulates of non-biological origin and do not stimulate active droplet formation like ballistospores.

### Wider environmental importance

Mushroom expansion is a hydraulic process that involves the osmotic inflation of tissues that expose gills and other configurations of spore-producing tissues. Mushroom-forming basidiomycetes are vital to the productivity of many forest ecosystems supported by heavy rainfall [21]. The behavior of spores revealed in this study suggests that a positive feedback mechanism may operate in which fungi whose growth is stimulated by rain, disperse massive quantities of spores that enhance precipitation. This hypothetical process is a consequence of a mechanism of spore discharge that occurs in 16,000 described species of mushrooms as well as basidiomycete yeasts and rust fungi that infect plants. There is no adaptive significance to the putative effect of spores on cloud formation. It is a consequence of the dispersal mechanism that happens to benefit the fungus beyond its effectiveness at distributing spores. If changes in climate reduce rainfall in tropical ecosystems, the resulting inhibition of fungal growth and spore release may exacerbate the frequency of droughts through this unexpected feedback loop.

## Supporting Information

S1 Video Video clip showing droplet expansion on basidiospore of *Russula aeruginea* at 102% RH. (MOV)

S2 Video Video clip showing droplet expansion on basidiospore of *Russula pulchra* at 102% RH. (MOV)
